# Preparation and Application of Bioshell Calcium Oxide (BiSCaO) Nanoparticle-Dispersions with Bactericidal Activity

**DOI:** 10.3390/molecules24183415

**Published:** 2019-09-19

**Authors:** Yoko Sato, Masayuki Ishihara, Shingo Nakamura, Koichi Fukuda, Tomohiro Takayama, Sumiyo Hiruma, Kaoru Murakami, Masanori Fujita, Hidetaka Yokoe

**Affiliations:** 1 Division of Biomedical Engineering, Research Institute, National Defense Medical College, 3-2 Namiki, Tokorazawa, Saitama 359-8513, Japan; ysato@ndmc.ac.jp (Y.S.); snaka@ndmc.ac.jp (S.N.); khf05707@nifty.com (K.F.); res337@ndmc.ac.jp (S.H.); 2 Department of Oral and Maxillofacial Surgery, National Defense Medical College, 3-2 Namiki, Tokorozawa, Saitama 359-8513, Japan; taka01@ndmc.ac.jp (T.T.); murakami@ndmc.ac.jp (K.M.); yokoe@ndmc.ac.jp (H.Y.); 3 Division of Environmental Medicine, Research Institute, National Defense Medical College, 3-2 Namiki, Tokorozawa, Saitama 359-1324, Japan; fujitama@ndmc.ac.jp

**Keywords:** scallop-shell powder, calcium oxides, dispersion, microbicidal activity, deodorization, cyro-SEM

## Abstract

Scallop-shell powder (SSP) heated at high temperature exhibits high pH and broad antimicrobial activity. Bioshell calcium oxide (BiSCaO) is an SSP composed mainly of calcium oxide. It is poorly water-soluble under alkaline conditions and the generated precipitate can plug spray nozzles. The aim of this study was to establish that BiSCaO dispersion caused no significant CaO loss and plugging of spray nozzles, and to evaluate its deodorization and microbicidal abilities and its ability to reduce the concentrations of NO_2_^−^ and NO_3_^−^. BiSCaO dispersions were prepared by mixing various concentrations of BiSCaO suspension, while phosphate compounds such as Na_3_PO_4_, Na_2_HPO_4_ or NaH_2_PO_4_ and the pH, average diameter, zeta potential, and form of the compounds with cryo-SEM were evaluated. We evaluated deodorization using tainted pork meat and microbicidal efficacy using contaminated suspension with normal bacterial flora. The concentration of NO_2_^−^ and NO_3_^−^ after mixing BiSCaO dispersion and pure water containing a high proportion of NO_2_^−^ and NO_3_^−^ were measured. BiSCaO dispersion formed with Na_2_HPO_4_, whose ratio to BiSCaO was 60%, showed a high pH (>12), a small particle diameter (>181 nm) and was stable for seven days. The BiSCaO dispersion showed higher deodorization and microbicidal activities than SSP-Ca(OH)_2_, which was mainly composed of Ca(OH)_2_. BiSCaO, but not SSP-Ca(OH)_2_, could reduce the concentration of NO_2_^−^ and NO_3_^−^ by more than 90% within 15 min. We developed a stable BiSCaO dispersion, and it had high deodorization and microbicidal efficacy. These activities of BiSCaO might result from the high pH caused by CaO hydration and a reduction activity causing active radical species.

## 1. Introduction

Various metal nanoparticles (MNPs) have been used in diverse fields, ranging from environmental remediation and biomedicine to catalysis, opt-electronic materials, and sensors [1,2]. MNPs comprising silver [1,3,4], copper oxide [1,2,5], zinc oxide [1,2,6], titanium oxide [2,7], and calcium oxide (CaO) [8,9] have strong antimicrobial activity against most microorganisms, including bacteria, fungi, and viruses. CaO nanoparticles have been synthesized by solution combustion methods using Ca(NO)_3_ and NH_2_CONH_2_ dissolved in water [10,11]. The proposed mechanism underlying the cytotoxicity of MNPs is considered to be at least partially due to the induction of reactive oxygen species (ROS), resulting in oxidative stress [12,13,14,15].

Calcium oxide produced from limestone (LiMCaO) is an important inorganic compound used in various industries as, for example, an adsorbent, toxic-waste remediation agent, and an alkalization agent. However, LiMCaO contains harmful impurities and has a dangerously high heat of hydration [16,17]. In contrast, scallop shells are a readily available source of CaO and are used as a food additive, as well as in plastering and paving materials. However, most scallop shells are considered to be industrial waste, and the shells accumulate on the shores of harvesting districts in Japan, causing serious problems such as offensive odors and soil pollution due to harmful materials and heavy metals leaching from the shells [18]. Heated scallop shell powder (SSP) is well known to exhibit potent microbicidal activity [19]. For example, SSP heated above 1000 °C and then ground shows broad microbicidal action against various viruses [20], bacteria [18,19], heat-resistant bacterial spores [18,21], fungi [22], and biofilms [12,23,24]. In addition, this material has served as an additive to prolong the shelf life of food products [19,24]. CaO is easily converted to Ca(OH)_2_ by hydration with water. CaO hydration generates a base and is considered to be one of mechanisms for the disinfection action of heated-SSP. For example, the disinfection activity of the CaO hydration reaction towards both total viable cells (TC) and coliform bacteria (CF) was higher than that of Ca(OH)_2_ or NaOH solutions at the same pH [24,25]. BiSCaO suspension (0.2 wt%) has been applied for the cleansing of contaminated wooden and pig skin pieces to remove both TC and CF, compared to the equivalent concentration of HOCl (pH 6.5) and NaClO (pH 9.5). BiSCaO suspension solution has higher disinfection activity than those of HOCl and NaClO in both TC and CF [25,26]. On the other hand, povidone–iodine and chlorhexidine gluconate have required more than 10-fold higher concentrations for the disinfection of BiSCaO [25,26]. Thus, BiSCaO has great potential to apply in medical care and the food industry.

Slurries of heated SSP (particle diameter range: 60–900 nm) are prepared by grinding shells heated to over 1100 °C in a wet bead-grinding mill [19] and suspending the powder in sterile saline. The main component of this heated shell powder slurry is calcium hydroxide (SSP-Ca(OH)_2_). Similarly, most commercially available heated shell powder products used as food additives are composed of Ca(OH)_2_. We used commercially available bioshell calcium oxide (BiSCaO, Plus Lab Co. Ltd., Kanagawa, Japan) in this study. According to the product instructions, BiSCaO contains over 99.6% CaO.

BiSCaO is poorly water-soluble under strongly alkaline conditions (pH > 12). Water suspensions of high concentrations of BiSCaO can result in a significant loss of CaO during handling and can plug spray nozzles [25,27]. Therefore, it is necessary to prepare dispersions containing stabilized CaO nanoparticles. Various concentrations of BiSCaO dispersions containing CaO NPs (160–300 nm) have been prepared by mixing BiSCaO with phosphate compounds such as Na_3_PO_4_, Na_2_HPO_4_, or NaH_2_PO_4_. In this study, we established conditions for generating BiSCaO dispersions using phosphoric acid and phosphate compounds by optimizing pH, particle form, particle size, and zeta potential. We then studied the deodorizing and disinfecting effects of the resulting BiSCaO dispersions and their NO_2_/NO_3_ reducing activity.

## 2. Results

### 2.1. BiSCaO Dispersion and Suspension with H_3_PO_4,_ Na_3_PO_4_, Na_2_HPO_4,_ and NaH_2_PO_4_

**Figure 1:**
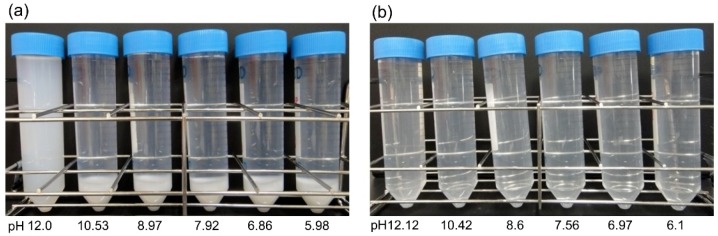
Addition to H_3_PO_4_, HCl to BiSCaO-6 suspension. Various amounts of H_3_PO_4_ (**a**) or HCl (**b**) were added to 2000 ppm (0.2 wt%) BiSCaO-6 water suspensions to adjust the pH to around 6, 7, 8, 9, 10.5, and 12.

Various amounts of phosphoric acid (H_3_PO_4_), hydrochloric acid (HCl), or sulfuric acid (H_2_SO_4_) were added to suspensions of heated SSP with an average particle diameter of 6 μm (BiSCaO-6) to adjust the pH. The results are shown in Figure 1 and Table 1. When adding 1 N H_3_PO_4_ to 0.2 wt% BiSCaO-6 suspension, the pH changed from 12.53 to 12 and dispersion without any precipitate was formed. The dispersion was stable without generating any flocculent and precipitation for at least seven days. Flocculation was formed from pH 10.5 to pH 6, and the flocculent completely dissolved to provide a clear solution at pH ≤ 5 (data not shown). In contrast, the addition of HCl or H_2_SO_4_ (Table 1) to 0.2 wt% BiSCaO-6 suspension generated neither a dispersion nor a flocculent. The precipitations were still formed at pH 12; however, they completely dissolved to provide clear solutions when the pH was lowered to pH ≤ 10.5.

**Figure 2:**
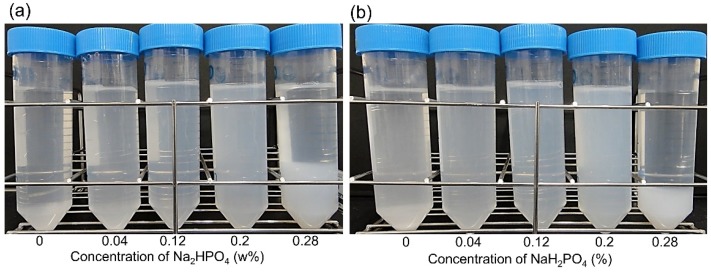
Formation of BiSCaO-6 dispersions and flocculants by the addition of Na_2_HPO_4_ or NaH_2_PO_4._ 0.04 wt%, 0.12 wt%, 0.2 wt%, and 0.28 wt% of Na_2_HPO_4_ (**a**) and NaH_2_PO_4_ (**b**) were added to 0.2 wt% BisCaO-6 suspension and rotary mixed.

Next, various amounts of Na_3_PO_4_ (Table 2), Na_2_HPO_4_, or NaH_2_PO_4_ (Figure 2, Table 2) were added to 0.2 wt% BiSCaO-6 suspension. Adding 0.28 wt% Na_3_PO_4_, Na_2_HPO_4_, or NaH_2_PO_4_ resulted in formation of flocculation, and adding 0.2 wt% still caused slight flocculation. On the other hand, adding 0.12 wt% Na_3_PO_4_, Na_2_HPO_4_, or NaH_2_PO_4_ resulted in dispersion without any precipitates and flocculates. Those dispersions were maintained for at least seven days. 

### 2.2. SEM Image of Dry BiSCaO-6 Powder and Nanoparticles in BiSCaO-6 Dispersions Formed by Adding H_3_PO_4_, Na_3_PO_4_, Na_2_HPO_4_, or NaH_2_PO_4_

The addition of H_3_PO_4_ (to pH 12), or 0.12 wt% Na_3_PO_4_, Na_2_HPO_4_, and NaH_2_PO_4_ to a 0.2 wt% BiSCaO-6 or BiSCaO (seven days) suspension resulted in a dispersion without precipitates and flocculates, the average particle diameter was 160–220 nm, and the zeta potential was in the range +33.6 to +35.9 one day after adjustment (Table 3). Those dispersions remained stable for at least seven days in terms of pH, average diameter, zeta potential, and phase form.

**Figure 3:**
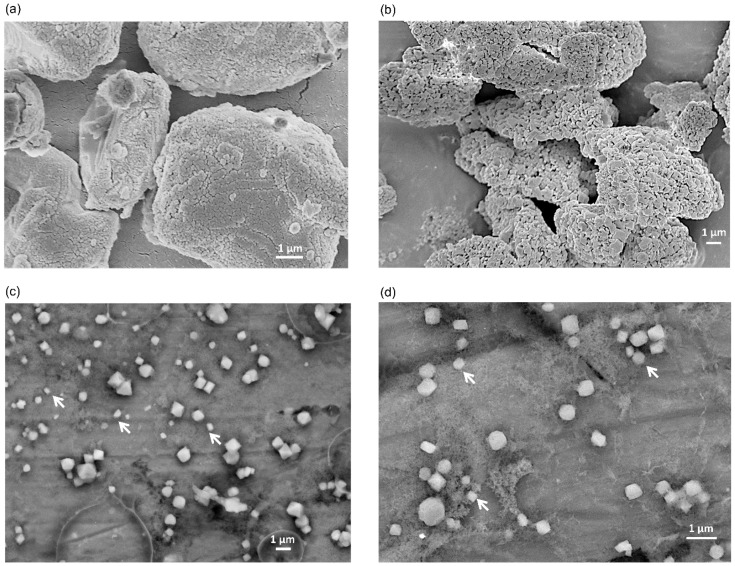
SEM images of BiSCaO-6 dry powder and dispersion. The surface structure of each dry powder of BiSCaO-6 immediately after opening at 10,000-fold magnification (**a**) and BiSCaO-6 placed under high humidity at 37 °C for seven days at 5000-fold magnification (**b**) were observed with SEM images of a field-resolved scanning electron microscope. Cryo-SEM observations were performed on BiSCaO-6 dispersions (0.2 wt% BiSCaO-6, 0.12 wt% Na_2_HPO_4_) two days after adjustment at 5000-fold magnification (**c**) and 10,000-fold magnification (**d**). Allows indicate nano particles.

We present the SEM images of BiSCaO-6 dry powder and dispersion adjusted with 0.2 wt% BiSCaO-6 and 0.12 wt% Na_2_HPO_4_ in Figure 3. BiSCaO-6 immediately after opening is a polymorphic powder with a wrinkled surface structure (Figure 3a), while BiSCaO-6 placed under high humidity at 37 ℃ for seven days to prepare BiSCaO-6 (seven days) exhibited a porous surface structure similar to that of nanoparticle aggregates (Figure 3b). Those observations suggested that hydration reaction on the surface of BiSCaO-6 may lead to the refinement of CaO crystals and rift and pore formations, which may promote the production of nanoparticles from microparticles in water dispersion. Indeed, Figure 3c,d indicated that CaO particles in BiSCaO-6 dispersion were nano scales.

### 2.3. Deodorization of Contaminated Minced Pork by BiSCaO Dispersions

**Figure 4:**
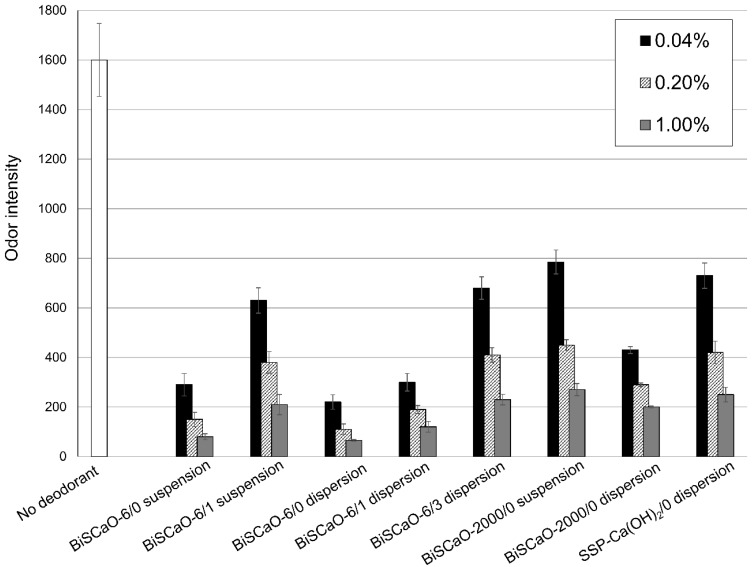
Efficacy of BiSCaO suspensions and dispersions for deodorizing contaminated minced pork. The indicated concentration and time after adjustment (/0: immediately after adjustment, /1: one day after adjustment, /3: three days after adjustment) of each BiSCaO suspension or dispersion was added to tainted pork meat on a petri dish, then sealed in a plastic bag for 1 h. The odor intensity was then measured using a handheld odor meter. The experiments were repeated three times and all provided results identical to the results shown.

We evaluated the deodorization efficacy of BiSCaO suspension and dispersion using tainted pork meat as a malodorous material. BiSCaO-6, BiSCaO-2000, and SSP-Ca (OH)_2_ suspensions (0.04 wt%, 0.2 wt%, and 1.0 wt%) were prepared. We chose Na_2_HPO_4_ as an additive to form dispersion because of the generation of smaller particles and added Na_2_HPO_4_ to each suspension. The ratio of Na_2_HPO_4_ to BiSCaO and SSP-Ca(OH)_2_ was 60%. We examined suspensions and dispersions immediately after adjustment (/0), one day after adjustment (/1), and three days after adjustment (/3), as shown in Figure 4. Tainted pork meat was mixed with each suspension or dispersion, then placed on a Petri dish and sealed in a plastic bag for 1 h. The odor intensity was then measured using a handheld odor meter.

BiSCaO-6/0 dispersion had the highest deodorization efficacy at each concentration tested and BiSCaO-6/1 and BiSCaO-6/3 dispersions were less efficient (Figure 4). A similar daily reduction for 0–3 days of deodorization efficacy by BiSCaO-2000/0 dispersion was observed (data not shown). In addition, SSP-Ca(OH)_2_ dispersion had a lower deodorization efficacy than BiSCaO dispersion.

### 2.4. Microbicidal Efficacy of BiSCaO Dispersions

We investigated the microbicidal efficacy of BiSCaO and SSP-Ca(OH)_2_ dispersions. A contaminated suspension with normal bacterial flora (total viable cells (TC) and coliform bacteria (CF)) was prepared by incubating remaining water in bathtub with 10% Dulbecco’s modified Eagle’s medium (DMEM) at 37 °C for 24 h [15,27]. In this incubation, the TC and CF cell count in the contaminated suspension increased from 100 ± 45 and 65 ± 30 to 6.8 ± 1.5 (×10^8^) CFU/mL and 6.6 ± 1.8 (×10^7^) CFU/mL, respectively. After preparing the following concentrations of 1200 (0.12 wt%), 400 (0.04 wt%), 133 (0.0133 wt%), and 44 (0.0044 wt%) ppm BiSCaO and SSP-Ca(OH)_2_ suspensions, dispersions were formed by adding 60% Na_2_HPO_4_ to BisCaO or SSP-Ca(OH)_2_ to each suspension. We examined dispersions immediately after adjustment (-/0), one day after adjustment (/1), and three days after adjustment (/3) as shown in Figure 4. Equal amounts of a dispersion and the contaminated suspension were mixed well and incubated at room temperature for 15 min; then, the number of colony-forming units (CFU/mL) per sample was determined. The CFU/mL of 600 ppm BiSCaO-6/0 dispersion was not detectable in both TC and CF, whereas some TC and CF remained viable at 200 ppm or lower BiSCaO-6/0 dispersion (Figure 4). BiSCaO/1 and BiSCaO/3 dispersions had lower microbicidal activities at all concentrations than BiSCaO/0 dispersion (Figure 4). SSP-Ca(OH)_2_ dispersion had lower microbicidal activity than BiSCaO dispersion at any concentrations.

### 2.5. Reduction of Aqueous NO_2_ and NO_3_ by BiSCaO

**Figure 5:**
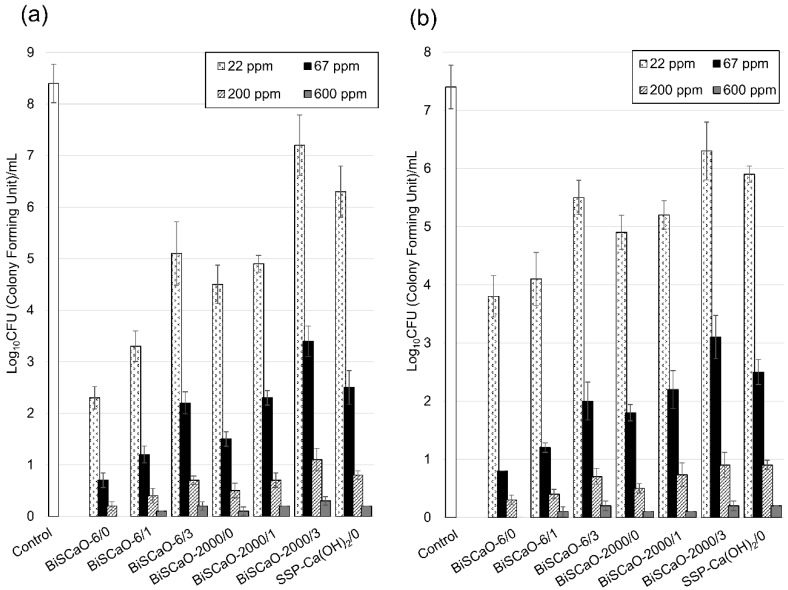
Microbicidal efficacy of BiSCaO dispersion. The number of colony forming units (CFUs) of (**a**) total viable microbe counts (TC) and (**b**) coliform bacteria (CF) in the contaminated suspension mixed with each BiSCaO were counted after gentle vortexing three times for 30 s and incubated at room temperature for 15 min (*n* = 4). Pure water as control of disinfectant was used as the control.

We studied the reduction in concentration of NO_3_^−^ and NO_2_^−^ by BiSCaO. The addition of 0.2 wt% BiSCaO-6, BiSCaO-2000 or LiMCaO powders to pure water containing NO_2_^−^(1.8 ppm) and NO_3_^−^(30 ppm) resulted in more than a 90% reduction of both NO_2_^−^ and NO_3_^−^ at room temperature within 15 min (Figure 5a,b). On the other hand, SSP-Ca(OH)_2_ and LiMCa(OH)_2_) did not reduce NO_2_^−^ or NO_3_^−^ concentrations over a period of 20 h. We incubated BiSCaO-6 and BiSCaO-2000 powders at 37 °C in the absence of desiccant for seven days before adding them to pure water containing NO_2_^−^ and NO_3_^−^. BiSCaO-6 (seven days) and BiSCaO-2000 (seven days) mildly reduced NO_2_^−^ by half over a period of 1 and 3 h, and reduced NO_3_^−^ by half over a period of 3 and 5 h, respectively.

**Figure 6:**
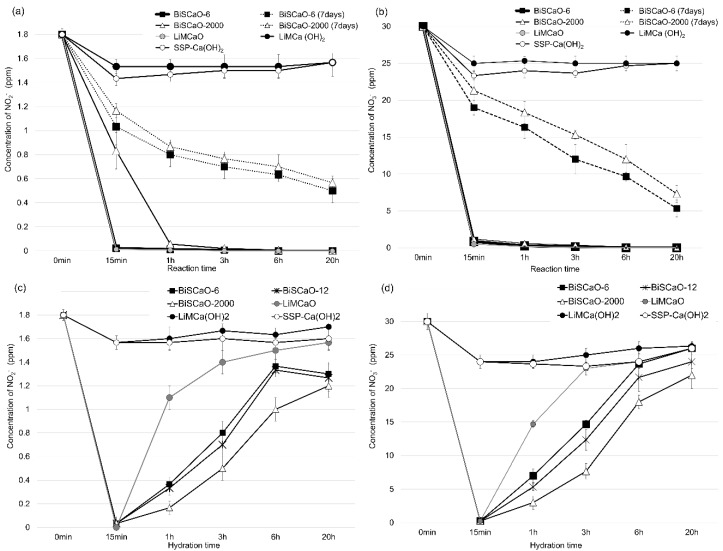
Decreasing NO_3_^−^ and NO_2_^−^ by BiSCaO, SSP-Ca(OH)_2_, LiMCaO, and LiMCa(OH)_2_. (**a**,**b**) The concentration of NO_3_^−^ and NO_2_^−^ at the indicated reaction times after adding 0.2 wt% BiSCaO-6, BiSCaO-2000 or LiMCaO powders to pure water containing 1.8 ppm NO_2_^−^ and 30 ppm NO_3_^−^ were measured. BiSCaO-6 (seven days) and BiSCaO-2000 (seven days) were incubated at 37 °C in the absence of desiccant for seven days before adding to pure water containing NO_3_^−^ and NO_2_^−^. (**c**,**d**) Each BiSCaO and LiMCaO powder was hydrated in pure water during several time periods, then added to pure water containing NO_3_^−^ and NO_2_^−^. The mixture was incubated at room temperature for 1 h and the concentration of NO_3_^−^ and NO_2_^−^ was measured.

Each BiSCaO and LiMCaO powder (0.4 wt%) was hydrated in pure water during several time periods, then added to pure water containing NO_2_^−^ and NO_3_^−^ and mixed. The mixture was incubated at room temperature for 1 h and the measured concentration of NO_2_^−^ and NO_3_^−^. BiSCaO-6, BiSCaO-12, BiSCaO-2000, and LiMCaO reduced the concentrations of both NO_2_^−^ and NO_3_^−^ by half in 1, 3, 4, and 5 h of hydration, respectively (Figure 6c,d). Their reducing activity decreased with hydration time. On the other hand, SSP-Ca(OH)_2_ and LiMCa(OH)_2_ exhibited no reducing activity throughout the experiment.

## 3. Discussion

Some scallop shell waste is used as a calcium supplement in food and in plastering and paving materials, but most is considered industrial waste and is piled along coastlines, causing environmental problems such as offensive odors and soil pollution due to the leaching of harmful materials from the shells [18,19,24]. Scallop shells are mainly composed of calcium carbonate (CaCO_3_), which is converted to calcium oxide (CaO) when heated above 1000 °C. Heated shell powder (SSP) exhibits broad antimicrobial action against viruses, bacteria, spores, and fungi [18,19,24].

Slurries of SSP nanoparticles (particle diameter range: 60–900 nm) are prepared by grinding shells heated above 1000 °C using a wet bead-grinding mill [19] and suspending the powder in sterile saline. The main component of this heated-SSP nanoparticle slurry is calcium hydroxide (Ca(OH)_2_). Most commercially available heated-SSP products used as food additives are composed of Ca(OH)_2_ (>90%) and not CaO (<5%). BiSCaO (Plus Lab Co. Ltd., Kanagawa, Japan) used in this study is commercially available and is prepared by heating at 1450 °C for 4 h, then by grinding using a dry super grinder (Nano Jetmizer NJ-300-D, Aishin Nano Technologies Co. Ltd., Saitama, Japan), followed by cooling in a vacuum chamber and vacuum packing. BiSCaO comprises over 99.6% CaO, the average particle diameter is about 6 µm, and its positive zeta-potential indicates that it is a uniform and fine powder.

Both BiSCaO and SSP-Ca(OH)_2_ are poorly water-soluble under alkaline conditions. The generated precipitates in suspensions of high concentrations of BiSCaO and SSP-Ca(OH)_2_ can result in a significant loss of CaO and the plugging of spray nozzles. A methodology is therefore needed to prepare BiSCaO and SSP-Ca(OH)_2_ dispersions without precipitates. We compared several phosphate compounds such as H_3_PO_4_, Na_3_PO_4_, Na_2_HPO_4_, or NaH_2_PO_4_ as an additive for dispersions. We found some conditions for dispersion which were maintained for at least one week. For the following experiments to study the deodorization and microbicidal efficacy of BiSCaO dispersion, we chose Na_2_HPO_4_ as an additive to form dispersion because of the generation of smaller particles in dispersion, and set the weight ratio of Na_2_HPO_4_ to BiSCaO as 60%. CaO particles in BiSCaO dispersion were nano scales with 100–300 nm.

We examined deodorizing and bactericidal activity of BiSCaO dispersion under conditions such as the type of BiSCaO (difference in particle size), concentration, and time from preparation of the dispersion to testing. Dispersion with smaller particle (BiSCaO-6), higher concentration, and immediately after adjustment showed highest deodorization and microbicidal activities against both TC and CF. BiSCaO-6 with smaller size has higher BET specific surface area (2.3 m^2^/g) than that of BiSCaO-2000 (0.8 m^2^/g) (data is not shown). It was expected that the difference of BET specific surface area of BiSCaO-6 and BiSCaO-2000 influenced deodorizing and microbicidal activities. BiSCaO-6 dispersion with immediately after adjustment showed higher deodorization and microbicidal activities than SSP-Ca(OH)_2_ dispersion with immediately after adjustment. Furthermore, BiSCaO-6 dispersion with three days after adjustment decreased their activities to the same level of SSP-Ca(OH)_2_ dispersion immediately after adjustment. These results suggested that CaO in dispersion has higher activities than Ca(OH)_2_ and CaO in dispersion was gradually hydrated with water and changed to Ca(OH)_2_ with decreasing deodorizing and microbicidal activities. Interestingly, BiSCaO-6 dispersion immediately and one day after adjustment showed higher deodorizing activities than BiSCaO suspension just after adjustment. This suggests that additive Na_2_HPO_4_ contributed the inhibition of hydration reaction from CaO to Ca(OH)_2_. The deodorization activity in this study considered to be derived a bactericidal activity to increased bacteria. However, a study showed that heated shell powders reduced formaldehyde [28], and the possibility of degradation activity of odorous components should be studied.

This study showed that BiSCaO and LiMCaO, but not SSP-Ca(OH)_2_ and LiMCa(OH)_2_, could reduce the concentration of NO_2_^−^ and NO_3_^−^ by more than 90% at room temperature within 15 min. These results suggest that CaO has reducing activity against both NO_2_^−^ and NO_3_^−^. The ability of BiSCaO and LiMCaO to reduce the concentration of both NO_2_- and NO_3_- decreased as the period of hydration with NO_2_^−^/NO_3_^−^ samples increased. However, the speed of decreasing activity with hydration of LiMCaO was faster than that of BiSCaO. These results suggested that as CaO was changed to Ca(OH)_2_ by hydration, the reducing activity of CaO decreased, and that BiSCaO was more stable than LiMCaO. In addition, when LiMCaO (20 wt%) was added to water and mixed, the suspension boiled (≥100 °C) within 0.5–5 min due to the heat of hydration of the reaction between CaO and water. In contrast, mixing BiSCaO with water resulted in a gradual and controlled increase in temperature (≤60 °C) over 10–80 min (data not shown). Thus, the heat generation of CaO in BiSCaO and LiMCaO may be correlated with a reducing effect. At any rate, further studies on the reducing effects of CaO molecules are required to make the mechanism clear.

CaO hydration generates a strong base and is the primary mechanism for the deodorization and microbicidal activities of BiSCaO dispersion. The CaO content of BiSCaO is much higher than that of SSP-Ca(OH)_2_, and this suggested that BiSCaO dispersion showed higher deodorizing and microbicidal activities than SSP-Ca(OH)_2_ because of the higher pH. However, our preliminary study showed that BiSCaO-6 exhibited higher activity than NaOH solution at the same pH (data not shown). This suggests that alkalinity alone is not responsible for the deodorizing and microbicidal property of BiSCaO. We found the possibility of a reducing activity of CaO with BiSCaO. Therefore, another possibility for the high disinfection activity of BiSCaO is that the OH^−^ concentration of the thin water layer formed around BiSCaO particles might be higher than in the bulk solvent [12,18]. Furthermore, it has been proposed that active radical species generated from MgO or CaO may also contribute to the stronger disinfection activity [12,18], as supported by a multi-parameter flow cytometry study conducted by Hewitt et al. [29]. Although high pH is certainly the main contributor to the deodorizing and microbicidal activity of BiSCaO, active radical species generated from BiSCaO may be an alternative microbicidal factor. The active radical species produced by MgO or CaO is poorly understood at present. Krishnamoorthy et al. [30] investigated the antibacterial activity of MgO, which, like CaO, is an alkaline earth metal oxide. They suggested that the anti-bacterial activity of MgO relies on the presence of defects or oxygen vacancies at the surface of the particles. Since MgO is easily hydrated and forms a surface layer of Mg(OH)2, it readily establishes surface-bound electron–hole pairs that can decompose into a surface-trapped electron and a localized hole state [31,32]. Further studies are required to understand the mechanism by which CaO exerts its microbicidal effects.

## 4. Materials and Methods

### 4.1. BiSCaO and LiMCaO Powders

Thorough cleaning scallop shell powders heated at 1450 °C for 2–6 h, and were natural cooling. The powders were pulverized by Nano Jetmizer (NJ-300-D, Aishin Nano Technologies Co. Ltd., Saitama, Japan) to produce BiSCaO-6. BiSCaO-6 (seven days) was prepared by placing BiSCaO-6 under high humidity at 37 °C for seven days. To prepare BiSCaO-2000, the cleaning scallop shells were baked at 1100 °C for 4 h, and subjected to natural cooling. The powders were pulverized by general-purpose rotor crusher (IMP-400, Seishin Enterprise Co. Ltd., Tokyo, Japan).

Table 4 showed the characterization of scallop shell powders we used in this study. BiSCaO had dry powder diameters of 3–9 (average 6.2) μm in BiSCaO-6, 2–7 (average 5.3) μm in BiSCaO-6 (seven days), and 200–2000 (average 500) μm in BiSCaO-2000 and were obtained from Plus Lab Corp., Kanagawa, Japan. SSP-Ca(OH)_2_ (Scallow) obtained from Kohkin Inst. Co. Ltd., Tochigi, Japan are dry powder diameter of 10–100 μm (average 46 μm). LiMCaO and LiMCa(OH)_2_ derived from limestone were purchased from FUJI FILM Wako Pure Chemical Corp., Osaka, Japan.

**Figure 7:**
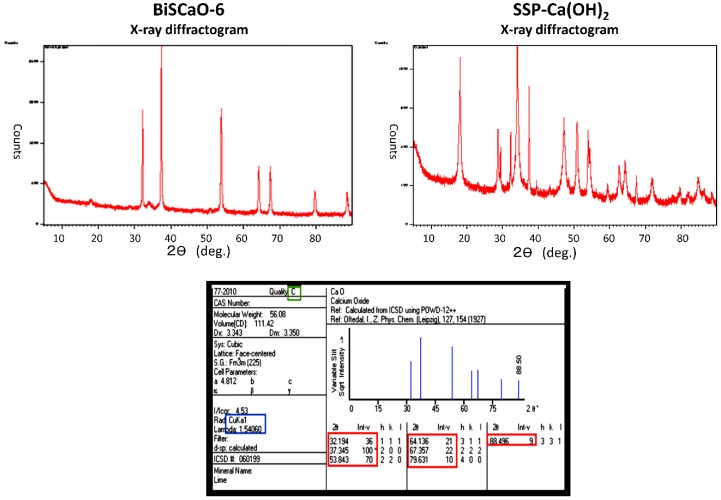
The contents of CaO and Ca(OH)_2_ in BiSCaO-6 and SSP-Ca(OH)_2_ were determined using X-ray diffractogram Contents of CaO in BiSCaO-6 and SSP-Ca(OH)_2_ was estimated 99.6% and <10%, respectively.

Average diameter of each dry powder ware determined using a particle size distribution measuring device (CILAS; Aishin-Nanotech Corp. Saitama, Japan). Average diameter and zeta potential of each particles in suspension or dispersion were determined using ELSZ-1000 (Otsuka Electronics Co. Ltd., Osaka, Japan). The contents of CaO and Ca(OH)_2_ in BiSCaO-6 and SSP-Ca(OH)_2_ were determined using an X-ray diffractometer system (Phillips X’Pert-PRO; Phillips Japan, Ltd. Japan) performed by the Kanagawa Institute of Industrial Science and Technology (Figure 7).

### 4.2. BiSCaO Water Suspensions and Dispersions with H_3_PO_4_, Na_3_PO_4_, Na_2_HPO_4_ or NaH_2_PO_4_

Addition to 0.2 g BiSCaO-6 to 100 mL of pure water, followed by rotary mixing, generated 0.2 wt% BiSCaO-6 suspensions. First, various amounts of phosphoric acid (H_3_PO_4_), hydrochloric acid (HCl) or sulfuric acid (H_2_SO_4_) were added to 0.2 wt% BiSCaO-6 suspensions to adjust the pH to around 6, 7, 8, 9, 10.5, 12, and 12.5. After the measurement of pH with a pH meter (F-70, HORIBA Ltd., Kyoto, Japan), the form and the proportion of dispersion or flocculation to total amount were observed. Next, 0.04 wt%, 0.12 wt%, 0.2 wt%, and 0.28 wt% of Na_3_PO_4_, Na_2_HPO_4_, or NaH_2_PO_4_ (FUJI FILM Wako Pure Chemical Corp.) were added to 0.2 wt% BisCaO-6 water suspension, rotary mixed, a7nd then evaluated for pH, form, and the proportion of layer separation with flocculation to the total amount.

### 4.3. Nanoparticles in BiSCaO Dispersions Formed Using H_3_PO_4_, Na_3_PO_4_, Na_2_HPO_4_ or NaH_2_PO_4_

Each 0.2 g of BiSCaO-6, BiSCaO-2000 or SSP-Ca(OH)_2_ to 100 mL of pure water, followed by rotary mixing, generated 0.2 wt% each water suspension. Then, 0.12 wt% Na_3_PO_4_, Na_2_HPO_4_, or NaH_2_PO_4_ was added to each suspension. The amount of H_3_PO_4_ were adjusted to be around pH 12. After rotary mixing, pH, average diameter, zeta potential, and form of each suspension were evaluated. Average diameter and zeta potential of particles were measured by ELSZ-1000 (Otsuka Electronics Co. Ltd., Osaka, Japan) [33,34].

For scanning electron microscope (SEM) images of dry powder, after osmium metal coating using a neo-osmium coater (Neoc-STB; Meiwafosis Co., Ltd., Tokyo, Japan), the surface structure of each dry powder was observed with SEM images of a field-resolved scanning electron microscope (JSM-6340F; JEOL Ltd., Tokyo, Japan). For cryo-SEM, samples were frozen in liquid nitrogen, then knife-cut and observed in JEOL JSM 7100F SEM (JEOL Ltd., Tokyo, Japan) under vacuum conditions at minus 90 degrees. The accelerating voltage was 10 KV, and the detection signal was a backscattered electron image.

### 4.4. Deodorization Activity of BiSCaO Dispersions

Added to 100 mL of pure water, 0.04 g, 0.2 g, and 1 g of BiSCaO-6, BiSCaO-2000, or SSP-Ca(OH)_2_, followed by rotary mixing, generated 0.04 wt%, 0.2 wt%, and 1.0 wt% for each water suspension. These water suspensions just after mixing were labeled as BiSCaO-6/0 suspension, BisCaO-2000/0 suspension, and SSP-Ca(OH)_2_/0 suspension. BisCaO-6/0, BiSCaO-2000/0 and SSP-Ca(OH)_2_/0 dispersions were prepared by rotary mixing Na_2_HPO_4_ in each water suspension. The ratio of Na_2_HPO_4_ to BiSCaO and SSP-Ca(OH)_2_ was 60%. Each water suspension or dispersion tested one day or three days after preparation were labeled as BiSCaO-6/1 suspension and BiSCaO-6/3 dispersion. Five grams of tainted pork meat was mixed with 10 mL of each water suspension or dispersion, then placed on Petri dishes and sealed in plastic bags (7 × 10 cm) for 1 h. The odor intensity was then measured using a handheld odor meter (OMX-SRM, Shinyei Technology Co. Ltd., Hyogo, Japan).

### 4.5. Microbicidal Efficacy of BiSCaO-Dispersions

To 100 mL of pure water, 120 mg, 40 mg, 13.3 mg, and 4.4 mg of BiSCaO-6, BiSCaO-2000, or SSP-Ca(OH)_2_ were added, followed by rotary mixing for 1 min, generating 1200, 400, 133, and 44 ppm for each water suspension. BisCaO-6, BiSCaO-2000, and SSP-Ca(OH)_2_ dispersions were prepared by rotary mixing Na_2_HPO_4_ in each suspension. The ratio of Na_2_HPO_4_ to BiSCaO and SSP-Ca(OH)_2_ was 60%. These dispersions just after mixing were labeled as BiSCaO-6/0, BisCaO-2000/0, and SSP-Ca(OH)_2_/0. Each dispersion tested one day or three days after preparation were labeled as BiSCaO-6/1 and BiSCaO-6/3. A contaminated suspension with normal bacterial flora (total viable cells (TC) and coliform bacteria (CF)) was prepared by incubating the remaining water in a bathtub with 10% DMEM at 37°C for 24 h [15,24]. Ten milliliters of each dispersion was added to 10 mL of the contaminated suspension, mixed well, and incubated at room temperature for 15 min; then, the number of colony-forming units per sample was determined. To count the number of colony-forming units (CFU), aliquots (1 mL of each mixture) were gently poured into individual Petri dishes containing pre-aliquoted portions of simple and easy dry medium for TC or CF (Nissui Pharmaceutical Co., Ltd., Tokyo, Japan) [25,31], and the plates were incubated for 24 h in a 37 °C incubator (Alp Co., Ltd., Tokyo, Japan).

### 4.6. Reduction of Aqueous NO_2_ and NO_3_ by BiSCaO

Regarding the evaluation of reaction time, 0.2 g BiSCaO-6, BiSCaO-2000 or LiMCaO powder was added to 100 mL pure water containing NO_2_^−^ (1.8 ppm) and NO_3_^−^ (30 ppm). BiSCaO-6 and BiSCaO-2000 powders, which were incubated at 37 °C for seven days in the absence of desiccant before adding pure water containing NO_2_^−^ and NO_3_^−^, were also evaluated. The levels of NO_3_^+^ and NO_2_^−^ were measured at 15 min, 1 h, 3 h, 6 h, and 20 h using a Pack Test for NO_3_/NO_3_-N and NO_2_/NO_2_-N with a Digital Pack Test-Multi SP (Kyoritsu Chemical-Check Lab., Corp., Tokyo, Japan) [35]. Regarding the evaluation of hydration time, 0.4 mg BiSCaO-6, BiSCaO-12, BiSCaO-2000, SSP-Ca(OH)_2_, LiMCaO, and LiMCa(OH)_2_ were hydrated in 100 mL pure water during several time periods: 15 min, 1 h, 3 h, 6 h, and 20 h. Then, those samples were added to 100 mL pure water, with NO_2_^−^ (3.6 ppm) and NO_3_^−^ (60 ppm) added to the pure water, and incubated at room temperature for 1 h, and the concentrations of NO_3_^−^ and NO_2_^−^ were calculated using a Pack Test.

## 5. Conclusions

We developed a BiSCaO dispersion by mixing with phosphate compounds without any precipitation, and flocculation caused no significant loss of the BiSCaO powder and plugging of spray nozzles after adding phosphate compounds such as Na_3_PO_4_, Na_2_HPO_4_, or NaH_2_PO_4_ to the BiSCaO suspension. The hydration reaction on the surface of BiSCaO-6 may lead to the refinement of CaO crystals and rift and pore formations, which may promote the production of nanoparticles from microparticles in water dispersion.

A dispersion of BiSCaO-6 with smaller size showed higher deodorization and microbicidal activities than BiSCaO-2000 with a larger size, SSP-Ca(OH)_2_, and BiSCaO suspension. Furthermore, BiSCaO, but not SSP-Ca(OH)_2_, could reduce the concentration of NO_2_^−^ and NO_3_^−^ in water solution within 15 min. These activities of BiSCaO might result from the high pH (>12) caused by CaO hydration and reduction activity, probably causing active radical species.

## Figures and Tables

**Table 1:** Changes in BiSCaO-6 suspensions by the addition of H_3_PO_4_, HCl_,_ or H_2_SO_4._

H3PO4	pH	12.53	12	10.53	8.97	7.92	6.86	5.98
	Dispersion (%)	0	100	0	0	0	0	0
	Flocculation (%)	0	0	20	17	16	15	15
	Form	Only precipitation	Dispersion without precipitation and flocculation	Layer separation with flocculation	Layer separation with flocculation	Layer separation with flocculation	Layer separation with flocculation	Layer separation with flocculation
HCl	pH	12.53	12.12	10.42	8.6	7.56	6.97	6.1
	Dispersion or Flocculation (%)	0	0	0	0	0	0	0
	Form	Only precipitation	Only precipitation	Solution	Solution	Solution	Solution	Solution
H2SO4	pH	12.53	12	10.42	8.97	7.95	6.89	5.95
	Dispersion or Flocculation (%)	0	0	0	0	0	0	0
	Form	Only precipitation	Only precipitation	Solution	Solution	Solution	Solution	Solution

The ratio of the layer of dispersion or flocculation to the total amount.

**Table 2:** Changes in BiSCaO-6 water suspensions by the addition of Na_3_PO_4_, Na_2_HPO_4,_ or NaH_2_PO_4_.

		0	0.04	0.12	0.2	0.28
Na_3_PO_4_	pH	12.55	12.55	12.55	12.25	12.1
	Layer sepa-ration with flocculation (%)	0	0	0	4	11
	Form	Only precipitation	Dispersion with precipitation	Dispersion	Dispersion with flocculation	Only flocculation
Na_2_HPO_4_	pH	12.55	12.53	12.5	12	11.95
	Layer sepa-ration with flocculation (%)	0	0	0	3	18
	Form	Only precipitation	Dispersion with precipitation	Dispersion	Dispersion with flocculation	Only flocculation
NaH_2_PO_4_	pH	12.55	12.49	12.45	12.05	11.65
	Layer sepa-ration with flocculation (%)	0	0	0	3	18
	Form	Only precipitation	Dispersion with precipitation	Dispersion	Dispersion with flocculation	Only flocculation

The ratio of the layer of dispersion or flocculation to total amount.

**Table 3:** Average diameter and zeta-potential of BiSCaO-6 particles in BiSCaO dispersions.

		Additive				
		Pure Water	H_3_PO_4_	Na_3_PO_4_	Na_2_HPO_4_	NaH_2_PO_4_
BiSCaO-6 (0.2 wt%)	pH	12.5	12	12.55	12.5	12.45
	Average diameter in dispersion (nm)	1060 (Supernatant)	160	200	181	190
	Zeta potential	+29.1	+35.9	+35.6	+33.6	+35.8
	Phase form	Suspension with precipitation	Dispersion			
BiSCaO-6 (seven days) (0.2 wt%)	pH	12.2	12	12.24	12.22	12.20
	Average diameter in dispersion (nm)	610 (Supernatant)	190	200	220	210
	Zeta potential	+29.0	+33.8	+33.9	+34.1	+33.6
	Phase form	Suspension with precipitation	Dispersion			

**Table 4:** Characterization of BiSCaO, SSP-Ca(OH)2, LiMCaO, and LiMCa(OH)2 used in this study.

		BiSCaO-6	BiSCaO-6 (Seven Days)	BiSCaO-2000	SSP-Ca(OH)2	LiM CaO	LiM Ca(OH)2
Average diameter of dry powder [Min-Max] (μm)		6.2 [3–9]	5.3 [2–7]	500 [200–2000]	46 [10–100]	18 [5–51]	12.5 [3.8–32]
Average diameter of supernatant of suspension (μm)		3.3	3.0	8.7	Not determined	4.5	3.1
Percentage of component (%)	CaO	99.6	20.1	97.0	1.8	10.3	8.2
	Ca(OH)2	0.2	73.3	2.2	97.6	85.2	89.7
pH of hydrogen ion concentration (0.2 wt%)		12.43	12.25	12.41	12.12	12.34	12.22
